# Brain-derived neurotrophic factor interacts with adult-born immature cells in the dentate gyrus during consolidation of overlapping memories

**DOI:** 10.1002/hipo.22304

**Published:** 2014-05-23

**Authors:** Pedro Bekinschtein, Brianne A Kent, Charlotte A Oomen, Gregory D Clemenson, Fred H Gage, Lisa M Saksida, Timothy J Bussey

**Affiliations:** 1 Department of Psychology, University of CambridgeCambridge, United Kingdom; 2 MRC and Wellcome Trust Behavioural and Clinical Neuroscience Institute, Translational and Cognitive Neuroscience Laboratory, University of CambridgeCambridge, United Kingdom; 3 Laboratory of Genetics, Salk Institute for Biological StudiesLa Jolla, California

**Keywords:** memory consolidation, dentate gyrus, BDNF, neurogenesis

## Abstract

Successful memory involves not only remembering information over time but also keeping memories distinct and less confusable. The computational process for making representations of similar input patterns more distinct from each other has been referred to as “pattern separation.” Although adult-born immature neurons have been implicated in this memory feature, the precise role of these neurons and associated molecules in the processing of overlapping memories is unknown. Recently, we found that brain-derived neurotrophic factor (BDNF) in the dentate gyrus is required for the encoding/consolidation of overlapping memories. In this study, we provide evidence that consolidation of these “pattern-separated” memories requires the action of BDNF on immature neurons specifically.

## INTRODUCTION

The ability to separate the components of memories into distinct complex memory representations that are unique and less easily confused has recently become of great interest to memory researchers. This is thought to occur by a computational process referred to as “pattern separation.” Computational models and experimental work have suggested that this crucial memory function may be localized to the dentate gyrus (DG) of the hippocampus (Gilbert et al., 1998; Leutgeb et al., 2007; McHugh et al., 2007) and, in particular, the adult-born immature neurons in this substructure (Aimone et al., 2009; Clelland et al., 2009; Nakashiba et al., 2012). However, little information is available regarding the molecular bases of this process. In this set of studies, we test the hypothesis that brain-derived neurotrophic factor (BDNF), acting on immature neurons, might be part of an essential mechanism underlying the consolidation of overlapping memories (Bekinschtein et al., 2011).

To test these specific ideas, we modified an established paradigm, spontaneous location recognition (SLR) (Ennaceur et al., 1997; Warburton et al., 2000), to allow parametric manipulation of the load on pattern separation (Bekinschtein et al., 2013). As pattern separation is thought to happen during encoding/consolidation stages of memory formation, the similarity of the to-be-remembered locations was varied during the encoding, rather than retrieval phase of the task. Different from other tests of pattern separation, the use of a continuous variable as a measure of performance yields sufficient data within a single trial to allow manipulations at different stages of memory. In contrast, previous tasks using discrete trial procedures require many trials to collect sufficient data, and thus such manipulations would have to be repeated an impracticable number of times.

Using this paradigm, we recently observed that BDNF in the DG is required during encoding/consolidation of “pattern-separated” memories (Bekinschtein et al., 2013). As immature neurons are thought to be involved in pattern separation (Clelland et al., 2009; Sahay et al., 2011; Nakashiba et al., 2012), it is possible that BDNF is interacting with these immature neurons during the pattern separation process; however, this idea remains to be tested. Therefore, in this study, we combined ablation of adult neurogenesis in the DG with local infusions of BDNF and we assessed pattern separation in our novel behavioral assay. We found that BDNF was able to enhance discrimination in control rats, but not in rats with diminished adult neurogenesis, indicating that BDNF is acting through immature neurons during this process.

## RESULTS AND DISCUSSION

**Figure 1:**
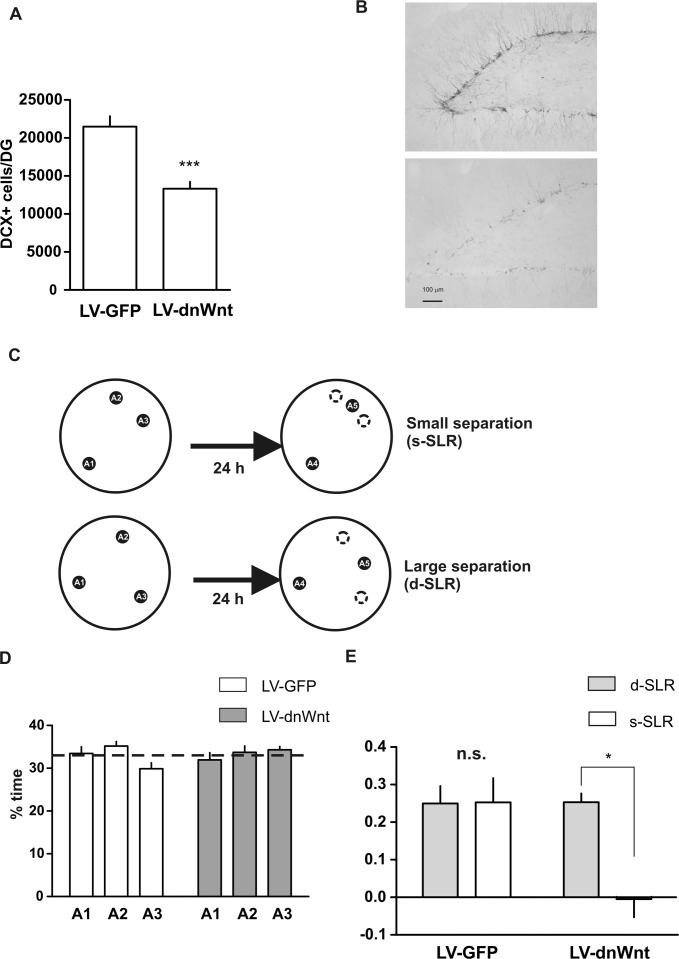
Adult-born immature neurons are required for pattern separation in the SLR task. (A) Absolute number of DCX+ cells in the DG of rats injected with a lentivirus-expressing dnWnt or a lentivirus-expressing GFP. ***P = 0.0002, *n* = 8. (B) Representative images of sections of a LV-GFP rat (top) and a LV-dnWnt rat (bottom) stained with an anti-DCX antibody. (C) Schematic representation of the SLR task for the s-SLR or d-SLR conditions. (D) Percentage of time exploring each of the locations during the sample phase of the SLR task. (E) Discrimination ratios for both groups of animals evaluated in the s-SLR and the d-SLR versions of the task (mean ± SEM). *P < 0.05, *n* = 8, Two-way repeated measures ANOVA followed by Bonferroni *post hoc* comparisons.

To test our hypothesis that BDNF modulates adult-born neurons in the DG during consolidation of overlapping memories, we modified the original SLR task (Ennaceur et al., 1997; Warburton et al., 2000) to be able to control the load on pattern separation during memory encoding (Bekinschtein et al., 2013). Briefly, our modified version of the task consisted of a sample (study) phase in which rats were exposed to three identical objects; two of them were close together and the third one was further away (Fig. 1C, top panel). In this way, the similarity of locations could be manipulated at the time of encoding, when pattern separation is thought to occur, rather than at retrieval, as in other tasks used to assess pattern separation (Gilbert et al., 1998; Clelland et al., 2009). During choice (test), the subject was exposed to two identical objects, one in a novel location between and equidistant from the two close ones explored during the sample phase, and the other one in its original location (Fig. 1C).

**Figure 2:**
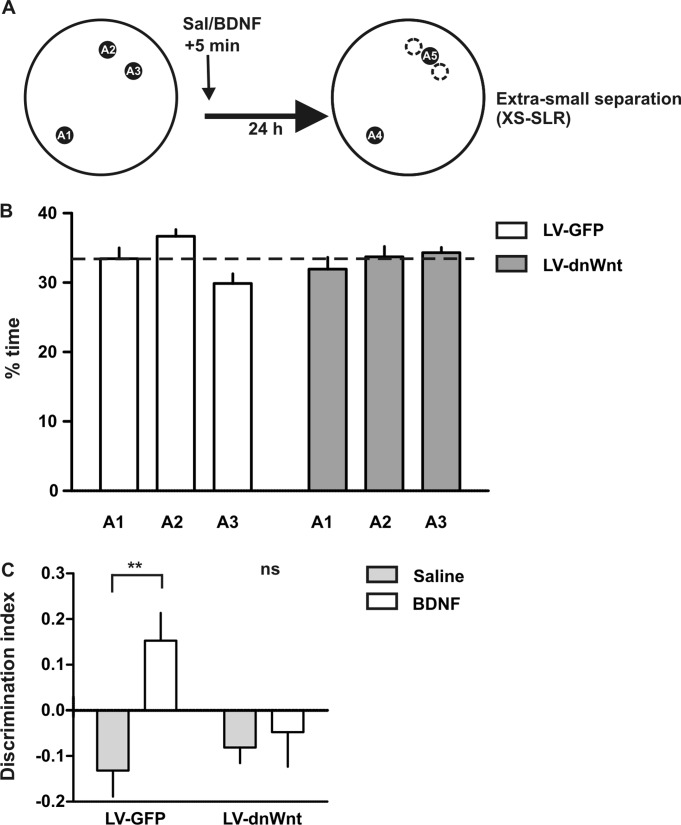
Adult-born immature neurons are necessary for BDNF effect on pattern separation. (A) Schematic representation of the xs-SLR. (B) Percentage of time exploring each of the locations during the sample phase of the xs-SLR task. (C) Effect of recombinant human BDNF (0.5 μg μL^−1^/0.5 μL side; rhBDNF) or saline, injected into the DG 5 min after the sample phase on performance during the choice phase in LV-GFP-injected or LV-dnWnt-injected rats. Discrimination ratios are expressed as mean ± SEM, **P < 0.01, *n* = 8, two-way repeated measures ANOVA followed by Bonferroni *post hoc* test.

We first tested whether blocking adult neurogenesis (Clelland et al., 2009; Nakashiba et al., 2012) would impair pattern separation in this paradigm. Such an impairment would be reflected in a failure of memory only when the load on pattern separation was high (when the locations are similar) but not when it was low (when the locations are dissimilar) (Clelland et al., 2009; Nakashiba et al., 2012). We used a lentiviral approach to specifically knockdown neurogenesis in the DG of adult male rats by inhibiting Wnt signaling, which is critically involved in the generation of newborn neurons, using a dominant-negative Wnt (dnWnt). Rats received stereotactical injections into the DG with either control lentivirus or lentivirus-expressing dnWnt to inhibit the generation of new neurons. This method was used successfully in the previous studies to reduce neurogenesis in rats and mice (Lie et al., 2005; Clelland et al., 2009; Jessberger et al., 2009). Stereotaxic injections allowed us to specifically target the DG, and thus minimizing involvement of extra-DG brain regions. The effects of this manipulation on behavior, in general, and on pattern separation in particular, have been shown to be the same as those obtained using other targeted methods such as irradiation (Clelland et al., 2009). The effects of this manipulation are also selective to behavioral conditions in which the load on pattern separation is high—behavior is not affected in internal control conditions in which task demands were identical but the load on pattern separation was low. Rats with normal levels of neurogenesis (LV-GFP) and rats with reduced levels of neurogenesis (LV-dnWnt) were tested in the SLR task. Animals were sacrificed after the behavioral testing and the levels of neurogenesis were determined by counting the doublecortin (DCX)-positive cells within the DG of control or LV-dnWnt-injected rats. LV-dnWnt rats showed a significant decrease in DCX+ cells compared to LV-GFP rats (Figs. 1A,B). For the behavioral experiment, the SLR task was run in two different ways by manipulating the separation between the locations to create two conditions with differing loads on pattern separation (Fig. 1C). In the “similar SLR” (s-SLR) condition, two of the locations were separated by a 50° angle and the third one by a 155° angle from the other two (small separation, Fig. 2C) and in the “dissimilar SLR” (d-SLR) condition, the three locations were separated by a 120° angle (large separation, Fig. 1C). We reasoned that if the rats needed to “pattern separate” the two close locations in the s-SLR condition but not in the d-SLR condition, then LV-dnWnt-injected rats should be impaired only in the s-SLR condition. Control and LV-dnWnt rats were tested either in the s-SLR or in the d-SLR condition; order of testing was counterbalanced with half of the rats from each group tested in the s-SLR first and half of them in the d-SLR first. LV-GFP rats and LV-dnWnt rats did not differ in the percent time spent exploring the objects during the sample phase and they spent an equal amount of time exploring each of the three objects. There was no main effect of treatment (*P* = 1.0, *n* = 8) or location (*P* = 0.92, *n* = 8) on the % time exploring the objects. (Fig. 1D). However, the results were different during the choice phase (Fig. 1E). A two-way repeated measures ANOVA showed a significant interaction of treatment (LV-dnWnt or LV-GFP injection) × separation (*P* = 0.037, *F*_(1,7)_ = 5.28). *Post hoc* contrasts revealed a significant effect of separation in the LV-dnWnt group (*P* < 0.05) but not in the control group. These results indicate that knocking down adult neurogenesis impairs memory retention in our novel spontaneous task only when the load for pattern separation during the sample phase is high (s-SLR).

Having identified BDNF as critical for encoding/consolidation of pattern-separated memories in DG (Bekinschtein et al., 2013), we could now begin asking questions about what cells might be involved in BDNF's mechanisms of action. At this point, we had determined, using a new paradigm and consistent with the previous studies (Clelland et al., 2009; Creer et al., 2010; Sahay et al., 2011; Tronel et al., 2012), that immature cells in the DG are required for pattern separation. Using this same paradigm, we showed that BDNF plays a role in pattern separation in the DG, specifically during encoding/consolidation of memory. In particular, BDNF was able to enhance pattern separation when injected exogenously into the DG (Bekinschtein et al., 2013). To tie these findings together, we asked the question: Is BDNF enhancing the consolidation of pattern-separated memories by acting on adult-immature neurons in the DG? We reasoned that if this were the case, BDNF should be able to enhance pattern separation in control rats but not in the LV-dnWnt rats with neurogenesis knockdown. To test this idea, we created experimental conditions in which animals were exposed to an “extra-similar separation” (xs-SLR). In the previous experiments, we found that control rats were not able to separate the two locations when separated by a 40° angle. The xs-SLR condition minimizes ceiling effects that might occur with the small separation. Both LV-GFP (control) and LV-dnWnt rats were tested in the xs-SLR condition and we evaluated the effect of infusing saline or rhBDNF immediately after the sample phase on memory retention. There was no main effect of treatment (*P* = 0.15) or location (*P* = 0.34) on the percent time exploring the objects. LV-GFP rats and LV-dnWnt rats did not differ in the percent time exploring the objects during the sample phase of the xs-SLR condition (Fig. 2B), indicating that, consistent with our results from Figure 1D, blocking neurogenesis did not modify exploratory activity. Memory retention was evaluated 24 h after the sample phase and we found a significant interaction of treatment (LV-dnWnt or LV-GFP) × drug (*P* = 0.016, *F*_(1,7)_ = 7.42, *n* = 8). Post hoc comparisons revealed a significant effect of rhBDNF infusion in control rats (*P* < 0.01, *n* = 8), but not in LV-dnWnt rats (Fig. 2C). These results indicate that adult-born immature neurons are required for BDNF-enhanced pattern separation in the SLR task and thus are required for memory consolidation of similar representations in the DG. The finding that it is the immature and not the mature cells that are critical is backed up by reports that while plasticity in immature neurons is required for pattern separation during contextual discrimination (Kheirbek et al., 2012), mature cells are not required and may indeed be more important for the complementary process of pattern completion (Nakashiba et al., 2012).

Although BDNF has been shown to increase survival of newborn neurons and increase neurogenesis (Scharfman et al., 2005; Rossi et al., 2006), it is unlikely that these processes are responsible for pattern separation in the present experiments. This is because the time course of the BDNF requirement for the task (minutes to hours) and development and incorporation of newborn cells into the circuits (weeks) are very different. Instead, the effect of BDNF on immature cells is an acute one. Immature adult-born neurons have been shown to be more excitable than mature neurons and also to have enhanced plasticity (Schmidt-Hieber et al., 2004; Ge et al., 2007), and hence they may respond more rapidly to the inputs of ambiguous spatial information in the DG. This enhanced response may be very sensitive to BDNF levels present in the hippocampus. Indeed, it has been shown that ablation of TrkB in progenitor cells has a significant effect on behavior and synaptic plasticity (Bergami et al., 2008). These results suggest that BDNF might be activating the TrkB receptor in immature neurons during pattern separation and that expression of BDNF might be the necessary stimulus for memory consolidation of similar representations to occur within the DG. TrkB activation might enhance plasticity by interacting with NMDA receptors. In fact, it has been shown that activated TrkB interacts with the NR2B subunit of the NMDA receptor increasing channel activity (Levine and Kolb, 2000; Mizuno et al., 2003; Xu et al., 2006). Another way in which BDNF could increase excitability is by decreasing GABA-dependent inhibition either by reducing excitability of fast-spiking interneurons (Holm et al., 2009) or by augmented internalization of GABA receptor subunits (Lu et al., 2010; Mou et al., 2011).

Our study provides an advance in our knowledge regarding the important process of pattern separation, by providing evidence that BDNF is one of the upstream signals that affects the plasticity of adult-born young neurons during this process. In addition, our results implicate these neurons in the process of memory consolidation, but only when the to-be-remembered information requires pattern separation. Until now, there was no information regarding how these cells were involved in performing this computational process and which plasticity signals were required for successful storage of distinct memories of similar spatial representations. It has been suggested that adult neurogenesis may have a more general function which includes, but is not restricted to, pattern separation, such as memory resolution (Aimone et al., 2011), or clearance of memories from the hippocampus (Kitamura et al., 2009). If these ideas are borne out, a target of future research will be to determine whether BDNF and adult-born neurons participate in the broader range of functions predicted by these theories.

## DETAILED EXPERIMENTAL PROCEDURES

### Subjects

The subjects were 16 Long–Evans rats (Harlan, San Diego, CA), weighing 250–300 g at the start of testing. The rats were housed on a reversed 12 h light/12 h dark cycle (lights on 19:00–07:00), in groups of two or four. All behavioral testing was conducted during the dark phase of the cycle. Rats were food deprived to 85–90% of their free feeding weight, except during recovery from surgery, where food was available *ad libitum*. Water remained available *ad libitum* throughout. All experimentation was conducted in accordance with the UK Animals (Scientific Procedures) Act 1986.

### Surgery and Cannulation

Rats were implanted bilaterally in DG of the dorsal hippocampus with 22-gauge indwelling guide cannulas. Subjects were anaesthetized with ketamine (Ketalar, 90 mg kg^−1^, i.p.) and xylazine (Rompun, 6.7 mg kg^−1^, i.p.) and placed in a stereotaxic frame (David Kopf Instruments, Tujunga, CA) with the incisor bar set at −3.2 mm. Guide cannulas (PlasticsOne™) were implanted according to the following coordinates, measured relative to the skull at bregma (Paxinos and Watson, 1998): anteroposterior, −3.9 mm; lateral, ±1.9 mm; and dorsoventral, −3.0 mm. The cannulas were secured to the skull using dental acrylic and three jewelry screws. Obturators, cut to sit flush with the tip of the guide cannulas and with an outer diameter of 0.36 mm, were inserted into the guides and remained there except during infusions. A screw-on dust cap kept the obturators in place. At the completion of each surgery, antibiotic powder (Acramide; Dales Pharmaceuticals, Skipton, United Kingdom) was applied. Animals were given at least 7 days to recover prior to drug testing.

### Virus Preparation

Lentivirus vectors were prepared as described previously (Lie et al., 2005). All viral stocks were diluted to and injected at 1 × 10^9^ transducing units (mL^−1^).

### Stereotaxic Injections

Sixteen male Long–Evans Rats (Harlan), 7–8 weeks old, were deeply anaesthetized with a ketamine/xylazine/acepromazine cocktail prior to surgeries. The rats were placed into a stereotaxic apparatus and received either a control GFP virus (LV-GFP) (*n* = 9) or a dnWnt virus (LV-dnWnt) (*n* = 9). A total of 6 μL of either lentivirus was injected, strategically placed over 16 injections spanning both hemispheres of the DG to allow for total coverage. Virus (0.3–0.4 μL) was injected slowly at each injection site for more than 1 min. After the procedure, animals were sutured and given a one-time injection of buprenex to help their recovery. Two animals, one control, and one dnWnt were excluded from the study after the DCX staining. The criteria were to exclude the data that deviated the *standard deviation* twice or more from the mean of the group. Cannulations and behavioral testing began 4 weeks after viral injections and rats were sacrificed for DCX staining immediately after testing ended, about 10 weeks after viral injections. All animal procedures were approved by the Institutional Animal Care and Use Committee at The Salk Institute for Biological Studies.

**Table:** Injection coordinates (from bregma):

	Anterior/posterior	medial/lateral	dorsal/ventral
1.	−2.4	±1	−4.1
2.	−3.2	±1.2	−4.1
3.	−4	±2	−3.7
4.	−4.8	±3	−3.8
5.	−5.4	±3.8	−4
6.	−5.4	±4.4	−7.2
7.	−6	±4	−4.2
8.	−6	±4	−7.4

### Infusion Procedure

Approximately, 5 weeks after viral injections, rats received bilateral infusions of human recombinant BDNF (0.5 μg μL^−1^/0.5 μL side; Byoscience) or saline. Bilateral infusions were conducted simultaneously using two 5-μL Hamilton syringes that were connected to the infusion cannulas by propylene tubing. Syringes were driven by a Harvard Apparatus precision syringe pump, which delivered 0.5 μL to each hemisphere for more than 2 min. The infusion cannulas were left in place for an additional minute to allow for diffusion. At least 3 days were allowed for washout between repeated infusions.

### Apparatus

The circular open field (90 cm diameter × 45 cm high) was made of black plastic. It was situated in the middle of a dimly lit room and it was surrounded by three proximal spatial cues and distal standard furniture. The open field floor was covered with wood shavings. A video camera was positioned over the arena and sample and choice phases were recorded on to DVD for later analysis. The objects used were either soda cans or beer bottles from which the label had been removed. They were fixed to the floor of the open field with Blu-tack™ and cleaned with 50% of ethanol solution between sample and choice trials. Positions varied according to the experiment, with objects always placed along a circumference 15 cm away from the wall and 30 cm away from the center of the arena.

### Behavioral Procedures

Each rat was handled for 3 days and then habituated to the arena for 10 min a day for 5 days before exposure to the objects. For the SLR task, after habituation, rats were exposed to three identical objects A1, A2, and A3, during a sample phase that lasted for 10 min. For the s-SLR, objects A2 and A3 were placed 50° apart (20.5 cm between them) and object A3 at an equal distance from the other two. For the d-SLR, objects A1, A2, and A3 were equidistant, 120° (49 cm between them) apart from each other. For the xs-SLR, A1 and A2 were separated by a 40° angle (15.4 cm between them). Twenty-four hours after the sample phase, rats were exposed to two new identical copies of the objects, named A4 and A5, for 5 min. New identical copies were used to prevent the use of olfactory cues. During this choice phase, object A4 was placed in a familiar location (same position as in the sample phase) and object A5 was placed in a novel location. For the s-SLR task, the novel location was defined as a position exactly in between the ones in which objects A2 and A3 were located during the sample phase (Fig. 1C, schemes). For the d-SLR task, object A4 was placed in a familiar location and object A5 in a position equidistant to the previous locations of A2 and A3 (Fig. 1C, schemes). One of the objects was always placed in a novel location. For the xs-SLR used in Figure 2, A1 and A2 were separated by a 40° angle (15.4 cm between them). The results were expressed as a discrimination ratio that was calculated as the time exploring the object in the novel location minus the time exploring the object in the familiar location over total exploration time ([*t*_novel_ − *t*_familiar_]/*t*_total_). For experiment shown in Figure 1, rats were tested twice in a counterbalanced order. For the experiment shown in Figure 2, each animal was also tested twice. Half of them in each group received saline and the other half rhBDNF in a counterbalanced order. Discrimination ratios were compared within subject two-way repeated measures ANOVA followed by Bonferroni *post hoc* comparisons.

### Histology

At the completion of behavioral testing, rats were anaesthetized by i.p. injection with 2 mL of Euthatal (Rhône Merieux) and perfused transcardially with phosphate-buffered saline (PBS), followed by 10% of neutral buffered formalin. The brains were removed and postfixed in formalin for at least 24 h before being immersed in 20% of sucrose solution until they sank. In total, 60-μm sections were cut on a freezing microtome encompassing the extent of the injector track.

### Immunohistochemistry (DCX staining)

To assess the level of neurogenesis in animals injected with the LV-dnWnt or the LV-GFP viruses, brains were prepared for immunohistochemistry for the microtubule-associated protein DCX, a marker for immature neurons, as described previously (Oomen et al., 2010). After perfusion, brains were cryoprotected by immersion in 20% of sucrose in 0.01 M of PBS, frozen, and cut in to 30-μm sections using a sliding microtome, and collected in 0.01 M of PBS. Sections were then incubated in primary antibody (polyclonal goat anti-DCX, Santa Cruz, 1:800) and signal amplification was accomplished by further incubation with biotinylated secondary antibody (horse antigoat [1:500, Vector]) and avidin–biotin enzyme complex (ABC kit; Elite Vectastain; 1:800). Subsequent chromogen development was performed with diaminobenzidine (20 mg 100 mL^−1^ TB, 0.01% H_2_O_2_). Stereological quantification (StereoInvestigator, Microbrightfield, Germany) of DCX-positive cells was done using a stage-controlled brightfield microscope (objective, 40×). Cells were counted at sites that were selected using systematic random sampling, in every tenth coronal section starting at bregma −2.1, in a total of six sections per animal. StereoInvestigator optical fractionator settings for DCX quantification were as follows: grid size, 200 × 80; counting frame, 50 × 50, which resulted in an average of 250 markers counted per animal.

### Data Collection

Exploration was recorded by the experimenter using a computer program written in Visual Basic 6.0 (Microsoft, USA). Two keys corresponded to the novel and familiar objects. Object exploration in both the sample and the choice phases was recorded by pressing the appropriate key at the onset of a bout of exploration and then pressing it again at the offset.
